# Risk assessment and reward processing in problem gambling investigated by event-related potentials and fMRI-constrained source analysis

**DOI:** 10.1186/s12888-014-0229-4

**Published:** 2014-08-10

**Authors:** Stephan F Miedl, Thorsten Fehr, Manfred Herrmann, Gerhard Meyer

**Affiliations:** Department of Neuropsychology and Behavioral Neurobiology, Center for Cognitive Sciences (ZKW), University of Bremen, Bremen, Germany; Center for Advanced Imaging - CAI Bremen, University of Bremen, Bremen, Germany; Institute of Psychology and Cognition Research, University of Bremen, Bremen, Germany; Department of Clinical Psychology, Psychotherapy and Health Psychology, Institute of Psychology, University of Salzburg, Salzburg, Austria; Department of Neurology II, Otto-von-Guericke-University Magdeburg, Magdeburg, Germany

**Keywords:** EEG, Addiction, Decision-making

## Abstract

**Background:**

The temporo-spatial dynamics of risk assessment and reward processing in problem gamblers with a focus on an ecologically valid design has not been examined previously.

**Methods:**

We investigated risk assessment and reward processing in 12 healthy male occasional gamblers (OG) and in 12 male problem gamblers (PG) with a combined EEG and fMRI approach to identify group-differences in successively activated brain regions during two stages within a quasi-realistic blackjack game.

**Results:**

Both groups did not differ in reaction times but event-related potentials in PG and OG produced significantly different amplitudes in middle and late time-windows during high-risk vs. low-risk decisions. Applying an fMRI-constrained regional source model during risk assessment resulted in larger source moments in PG in the high-risk vs. low-risk comparison in thalamic, orbitofrontal and superior frontal activations within the 600-800 ms time window. During reward processing, PG showed a trend to enhanced negativity in an early time window (100-150 ms) potentially related to higher rostral anterior cingulate activity and a trend to centro-parietal group-differences in a later time window (390-440 ms) accompanied by increased superior-frontal (i.e., premotor-related) source moments in PG vs. OG.

**Conclusions:**

We suggest that problem gambling is characterized by stronger cue-related craving during risk assessment. Reward processing is associated with early affective modulation followed by increased action preparation for ongoing gambling in PG.

**Electronic supplementary material:**

The online version of this article (doi:10.1186/s12888-014-0229-4) contains supplementary material, which is available to authorized users.

## Background

Pathological gambling is associated with loss of control and continued gambling in spite of negative consequences. In the Diagnostic and Statistical Manual of Mental Disorders V (DSM V [1]) it is classified as behavioral addiction with a lifetime prevalence of 0.5-1% [2]. Pathological gambling shares core features with substance addictions [3,4], such as loss of control and heightened attention for gambling-related situations. As context specific environment seems to play a crucial role in the maintenance of pathological gambling behavior similar to addiction [5], we applied an experimental design with a quasi-realistic blackjack game scenario [6] to increase ecological validity of the procedures. We focused on studying the temporal dynamics of regional brain activity underlying pathological gambling behavior by integrating EEG data source analysis with prior information of regional brain activity reported in a recent fMRI study [6], where problem gamblers (PG) compared to occasional gamblers (OG) showed higher inferior frontal, superior temporal, and thalamic activation during high-risk vs. low-risk assessment and enhanced fronto-parietal activation during reward processing. Especially, an ecologically valid experimental design, rich of gambling cues [7,8], might be linked to modulation of electrophysiological components in gamblers as problem gamblers showed stronger high-risk taking behavior and enhanced positive reward-related error-related negativity after successful high-risk hit decisions in a blackjack game [9]. This is in line with an involvement of medial frontal neural generators during exhibition of reward hypersensitivity in gamblers [10], economic choices [11], error detection [12] and deviations of motivational predictions [13]. Interestingly, stronger positive feedback negativity was linked to striatal source activity [14]. Furthermore, the absence of selective feedback negativity to near vs. full losses in PG [15] might reflect near losses to be not as punishing for PG as for controls, a finding that is in accordance to poor error processing in excessive computer gamers [16].

Two studies linked ERP and fMRI measures of reward within a single sample [17,18]. Carlson et al. [18] showed that fMRI activation in the mesocorticolimbic reward circuit was positively correlated with the feedback negativity in a win lose comparison, which fits to an enhancement of both anterior cingulate cortex (ACC) activation and medial frontal negativity during reward prediction violation [17]. Additionally, smokers demonstrated heightened N1/P1 amplitude during processing of addiction-related words, which was proposed to reflect increase in attention or early content-related and potentially automated addiction-cue-related information processing [19]. Moreover, P3/slow wave component was reported to be enhanced for cues of alcohol [20], smoking [21], cocaine [22], and heroin [23] in populations of substance-dependent individuals as well as for computer game related-cues in excessive computer gamers [24] and gambling related cues in gamblers [25]. Importantly, slow wave amplitude was sensitive to motivational relevance [26] as well. However, the here listed EEG studies were not conceptualised to obtain data for the analyses of underlying neuronal generators of the respective ERPs. Integrating ERP and fMRI data by fMRI constrained source analysis demonstrated an improvement of spatial and temporal information [27,28], which in our case should reveal more detailed information about temporal sequence of local brain activity during risk assessment and reward processing in problem gambling.

The present study uses information about activation foci revealed by a former fMRI study [6] to provide regional constraints for source models to be applied on EEG data measured for the present study similar to recent work of Trautmann-Lengsfeld et al. [29]. This should enhance the validity of respective source models evolved to explore differences in spatio-temporal dynamics of risk assessment and reward processing in PG and OG in an exploratory manner. During risk assessment it was expected that assessment of high-risk situations should be reflected by strengthening of gambling-cue-driven electrophysiological modulations in PG with underlying addiction-cue-related orbitofrontal sources [30]. At the moment of reward processing, we assumed early modulations of ERPs due to differences in attentional processing between PG and OG [19,31]. During later time windows, especially PG were expected to produce enhanced ERPs related to centro-parietal generators [32] triggered by enhanced cognitive processing of motivationally relevant win situations.

## Methods

### Study participants

The EEG study group consisted of 12 healthy male OG and 12 male PG (slot-machine gamblers). All participants were right handed, except for one ambidexterity in the PG group (modified Edinburgh Handedness Questionnaire); [33], and did not participate in the previous fMRI study using the same experimental task [6]. Both groups did not significantly differ in age and smoking behavior, and frequency of gambling was significantly higher in PG compared to OG (z = -4.5, p < 0.01; see Table 1).

**Table 1:** **Demographic and clinical data of PG and OG (mean ± standard deviation)**

	**PG (n = 12)**	**OG (n = 12)**	
Age	33.8 ± 7.8	35.8 ± 9.5	t[1,22] = 1.17, p = 0.58
Number of smokers per group	11	8	z = -1.48, p = 0.3
DSM IV	5.9 ± 2	0.9 ± 0.8	z = -4.20, p < 0.01
SOGS	9.7 ± 3.8	0.9 ± 0.8	t[1,22] = 23.37, p < 0.01
KFG	33.4 ± 10.9	3.8 ± 3.1	t[1,22] = 17.23, p < 0.01
Percent of income spent on gambling	64.6 ± 61.4	2.6 ± 3.2	t[1,22] = -3.65, p < 0.01
Blackjack frequency	< 1 time/month	< 1 time/month	z = -0.95, p = 0.4
Frequency of overall gambling behavior	> 3 times/week	≤ 3 times/month	z = -4.48, p < 0.01

The study group was restricted to male participants only as the prevalence of PG in men is reported to be two times higher than in women [34]. Participants were recruited through advertisements and were familiarized with the gambling environment in the laboratory. Prior to enrollment into the study, all participants underwent a structured psychiatric interview. No participant did report a history of psychiatric or neurological illness or regular drug use or reported a history of psychotropic medication. In the PG group, four participants were presented with a diagnosis of problem gambling (three or four criteria); [35] and eight participants had a diagnosis of pathological gambling (≥5 criteria) according to DSM IV. Participants were assessed with the German gambling questionnaire “Kurzfragebogen zum Glücksspielverhalten” (KFG); derived from 20 items as developed by “Gamblers Anonymous”; [36]. All PG scored between 19 and 51 points (threshold for PG is set at 16 points); OG scored ≤ 10. In addition, all participants were evaluated with a German version of the South Oaks Gambling Screen (SOGS); [37]. Participants who scored ≥ 5 points were classified as “probable pathological gamblers”. All PG scored between 5 and 16 points on the SOGS; OG scored ≤ 2 (see Table 1). The study protocol was designed according to the Code of Ethics of the World Medical Association (Declaration of Helsinki, 1984) and was approved by the local ethics committee of the University of Bremen. All participants were informed about the procedure and gave written informed consent to participate.

### Experimental design

**Figure 1:**
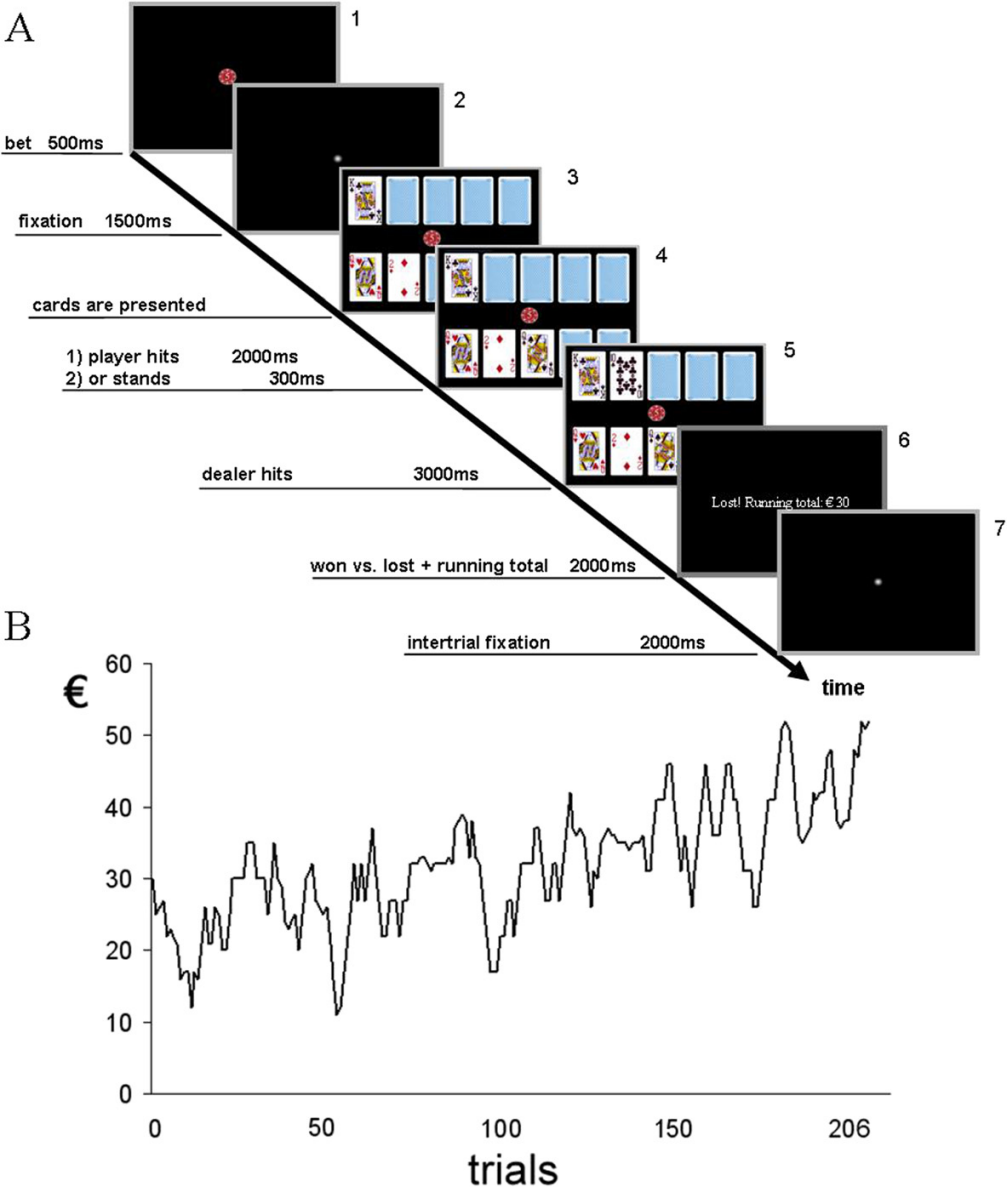
**Trial sequence and course of wins and losses. (A)** Trial description of task elements in a quasi-realistic blackjack scenario, **(B)** pre-determined win and loss course of the game.

The experimental blackjack task applied here (Figure 1) was identical to a version previously used in an fMRI study of our group [6] and consisted again of 206 trials. Fifty low-risk trials provide gaming situations in which the player started with 12 or 13 points against the dealer’s 7, 8, 9, or 10 points. Participants were informed that they would play against the computer. Fifty high-risk trials consisted of situations providing the player with 15 or 16 points and the dealer with 7, 8, 9, or 10 points. The probability of losing while drawing a card [P(lose|hit)] over all low-risk trials was 0.34, and 0.56 over all high-risk trials. The trials were designed in a way that - according to the blackjack basis strategy [38] - in all high-risk and low-risk situations a hit was more advantageous for the player than a stand ([P(lose|stand] = 0.77). Fifty fill-trials were composed of cards with pictures and numbers with no relation to the blackjack game, which potentially serve as low-level baseline condition in further analyses not reported here. Furthermore, we included 56 validity-trials, consisting of aces (1 or 11 points), and starting-situations with 14, 17, 18, 19, 20 or 21 points for the player. These validity-trials should help to simulate a quasi-realistic blackjack scenario. The bet was fixed at € 5 in low-risk and high-risk trials, and at € 1 in validity-trials. All trial elements were presented against a black background. A trial started with a chip representing a fixed bet (€ 1 or € 5; frame 1, see Figure 1A) for 500 ms (millisecond), followed by a white fixation point for 1500 ms (frame 2). Thereafter, three cards were presented for a maximum of 6000 ms; on the upper part of the screen one card for the dealer and on the lower two cards for the player (frame 3). Within this period the player had to decide whether he wanted to take another card (“hit”; left button click; index finger) or to stand (“no further card required”; right button click; middle finger, frame 4). Thereafter, the dealer took cards according to the official blackjack rules (the dealer has to hit until his total was 17 or higher). Dependent on the player’s response (hit or stand), the dealer started to take another card 300 ms after the player decided to stand (stand response), and 2000 ms after the player’s hit (hit response). The end of the round was presented for 3000 ms (frame 5), followed by a 2000 ms information screen displaying the running total of the player (frame 6) and a 2000 ms inter-trial fixation point (frame 7). Before the EEG acquisition session all participants were asked to perform 10 minutes of practice trials. In contrast to official blackjack rules the player was allowed to hit or to stand only one time per round.

At the beginning of the game, each player started with a balance of € 30. All study participants were informed that they might lose their starting balance and that they would receive the entire balance in cash at the end of the experiment. Wins and losses followed a predetermined course independent from the player’s decisions (see Figure 1B). Trials were presented in a pseudo-randomized non-stationary probabilistic sequence [39]. Participants lost 50 percent of the high-risk trials and 50 percent of the low-risk trials, and always finished the game with a total amount of € 52.

### EEG data acquisition

EEG data were recorded from 64 Ag-AgCl scalp electrodes placed according to the extended internationally standardized 10/20 system. The EEG signal was amplified (REFA multi-channel system; TMS international) and digitized with a sampling rate of 512 Hz (average-referenced). The ground electrode was located on the lower left cheek. Impedance per channel was kept below 15 kΩ. Vertical and horizontal electrooculographic (EOG) recordings were obtained from four additional Ag-AgCl surface electrodes (above and below the right eye; lateral to both eyes). Individual head electrode positions were digitized with Zebris Motion Analyzer System (CMS 20; Zebris Medical GmbH).

### EEG data analysis

The analysis of EEG data was restricted to low-risk and high-risk trials. In addition, we focused on two different time periods in the trials: 1) risk assessment, and 2) win or lose situations. For the first time period the ERPs were locked to the time point, at which the cards were presented (Figure 1A, frame 3). For the second period ERPs were locked to the time point of the dealer’s hit (Figure 1A, frame 5). In cases where the player hit and got 21 (win) or over 21 (lose) ERPs were time-locked to the moment when the player hit (Figure 1A, frame 4).

BESA® software (version 5.1.8.10; MEGIS Software GmbH; Munich Germany) was used to analyze EEG data. Artifacts were detected with the artifact scan tool implemented in BESA®, where sweeps at every channel with amplitudes above 100 μV were rejected. Additionally, single individual data was visually inspected for artifacts and channels were interpolated if necessary (the number of interpolated channels on single subject averages was kept below four). Eye movements were averaged separately for blinks and each movement direction and the resulting topographies were used as prototypic templates for a spatio-temporal correlation with the EEG data. After artifact rejection 87.4% of all high-risk and 85.3% of all low-risk trials were used for analysis. Data were high-pass filtered (0.01 Hz) prior averaging. For stimulus-locked analyses data were averaged from stimulus-onset to 1500 ms post-stimulus onset. ERP-data were baseline-corrected to the mean amplitude of the 100 ms pre-stimulus period. After averaging a low-pass filter of 30 Hz was applied and evoked potentials were visually inspected, and time windows (for the phase of risk-assessment: 380-420 ms and 600-800 ms post-stimulus onset; for the phase of reward processing: 100-150 ms and 390-440 ms post-stimulus onset) were defined for further analyses. To determine the respective ERP-time epochs for mean amplitude calculation, we inspected the ERP overlay plots of the grand average differences waves (separately for high vs. low risk and win vs. lose for OG minus PG) for peak activations (see Additional file 1: Figure S1 and Figure S2 red and yellow boxes). The late time window during reward processing was chosen around the peak in the electrode overlay plot and global field power according to the time window for the phase of risk assessment. Mean amplitude values were calculated for the defined time windows for each individual. Topographical analyses of mean amplitude values were performed separately for each ERP time interval including 15 approximately equidistant distributed electrode positions (F7, F3, Fz, F4, F8, T7, C3, Cz, C4, T8, P7, P3, Pz, P4, and P8).

### Statistical analysis of EEG data

For the phase of risk assessment, a four-way repeated measures ANOVA was calculated, including the within-subject factors ANTERIOR-POSTERIOR (AP, 3 levels: Frontal, central, and posterior electrodes), LATERALITY (LAT, 5 levels: From right to left scalp electrodes), RISK (high-risk, low-risk) and the between-subject factor GROUP (PG, OG). Analyzing the ERP time interval and factor composition during the phase of reward processing was identical as compared to the analysis of risk assessment, but with the factor REWARD (win, lose) instead of RISK. Main effects and significant interactions were corrected for sphericity violation where necessary. Significant four-way interactions legitimated exploration of single electrode-effects by subsequent t-tests. Two-way interactions including the factors GROUP and REWARD or three-way interaction effects including the factors GROUP, REWARD and ANTERIOR-POSTERIOR or LATERALITY were further explored with ANOVAs for each single electrode position. In case of significance (p < 0.05) or trend to significance (p ≤ 0.1), post-hoc analyses (t-tests) were calculated.

### FMRI constrained source analysis – model forming

Two separate source models were created for risk assessment and reward processing applied to 64 EEG-channel data. Regional sources (RS) were seeded for risk assessment according to fMRI activation foci [6] revealed by the group interaction contrast (high-risk PG > low-risk PG) > (high-risk OG > low-risk OG) and respectively (win PG > lose PG) > (win OG > lose OG) for reward processing as the main focus was on the differences between groups. Applying fMRI constrained source analysis is grounded in a combination of temporal and spatial dynamics while including prior information of fMRI activation foci to improve the spatial validity of the model [40,41]. Source waveforms were calculated using a four-shell spherical head model, which considered characteristics of the brain like, conductance, bone, cerebrospinal fluid, and scalp [28], and a regularization constant of 1 percent for the inverse operator to reduce the interaction between sources. A RS consists of three equivalent current dipoles with identical location but reciprocally orthogonal orientations [28,42]. As the activity of RS is hardly sensitive to small differences between the modeled location of active brain regions and individual anatomical location [28,43], the obtained source waveforms for the fMRI seeded sources should be quite robust despite including different participants in experiment 1 (fMRI) and experiment 2 (EEG). For risk assessment a multiple source model on group differences (PG vs. OG) of ERP difference waves (high-risk vs. low-risk) was applied. For reward processing the multiple source model was applied on group differences (PG vs. OG) of ERP difference waves (win vs. lose). The fMRI contrast for risk assessment [(high-risk PG > low-risk PG) > (high-risk OG > low-risk OG)] resulted in three activation foci seeded as RS (RS 1: right superior temporal gyrus, RS 2: right inferior frontal gyrus, RS 3: right thalamus; see Additional file 1: Figure S1), which were fixed according to their location in the source model. Furthermore, the fMRI contrast for the reward processing [(win PG > lose PG) > (win OG > lose OG)] yielded three activation foci seeded as RS (RS 1: right superior frontal gyrus, RS 2: left superior parietal lobe, RS 3: left anterior cingulate gyrus; see Additional file 1: Figure S2), which were also fixed according to their location in the source model. To avoid reciprocal interaction, so-called “crosstalk” between sources with a distance of less than 30 mm, they were averaged according to the nearest neighbor method [27]. This was necessary for the two parietal sources during reward processing, where the coordinates of the two sources were averaged. The new averaged source (see Additional file 1: Figure S2, RS 2) was located within the two centimeter range from its original fMRI peak locations. The averaging of the coordinates of neighboring sources is justified by the integrative nature of RS in a multiple discrete source model. The reason is that errors in the corresponding center location smaller than two centimeters do not sufficiently influence source waveforms, as long as the distances between the different sources are larger [27,40]. Additionally to the three fixed RS, a sequential fitting procedure was applied in BESA for risk assessment to reduce residual variance of the model. The time windows for the phase of risk assessment were defined around the first two peaks in the global field power curve. As there was no additional clear peak in the global field power curve a third long time window (500-940 ms) was defined (see Additional file 1: Figure S1B red boxes). For the 80-160 ms time window two sources, RS 4 and RS 5, were fitted. In the 360-430 ms time window, again two sources, RS 6 and RS 7, were fitted, while RS 4 and RS 5 were switched off. Furthermore, for the 500-940 ms time window two additional sources were fitted, RS 8 and RS 9, while RS 4-7 were switched off. The model for risk assessment, therefore, consisted of 9 RS and explained a variance of 93.4 percent (see Additional file 1: Figure S1). For reward processing, additional to the two fixed RS, a sequential fitting procedure to reduce residual variance was performed. The time windows for the phase of reward processing were defined around the first peak in the global field power. As there was no additional clear peak and a constant high level of global field power a second long time window (200-1000 ms) was defined (see Additional file 1: Figure S2B red boxes). For the 80-160 ms time window, RS 3-5 were additionally fitted, and for the 200-1000 ms time window, RS 6-11 were fitted, while RS 3-5 were switched off. The model for reward processing included 11 RS, which explained 94.2 percent of variance (see Additional file 1: Figure S2).

### fMRI constrained source analysis – data analysis

The obtained source models were applied on single individual data [difference ERP-waves for the difference between high-risk and low-risk and for the difference between win and lose]. The root mean square [RMS; the square root of the mean of the added and squared power (in nA/m) of three orthogonally oriented equivalent current dipoles at the same location] of each RS was calculated for each time point resulting in respective source moment curves. To explore the spatio-temporal dynamics of all RS, mean RMS values for relevant time-windows of the ERP-analysis for each RS and each study participant were calculated. Repeated measures ANOVAs on mean-RMS values were performed separately for risk assessment (380-420 ms and 600-800 ms time-window) and reward processing (100-150 ms and 390-440 ms time-window) including within-subjects factor REGIONAL SOURCE (RS: 9 levels during risk assessment; during reward processing (5 levels: 100-150 ms) and (11 levels: 390-440 ms)), and the between-subject factor GROUP (PG, OG). The number of sources included in the analyses was chosen according the state of the successive source model (the number of sources fitted to the model up to the analysed time-window). Significant (p < 0.05) or trend to significant (p ≤ 0.1) main effects of the GROUP * RS interactions were further explored with post-hoc analyses (t-tests). In case of not significant or trend to significant GROUP * RS interactions, the number of sources were limited to the number of active sources.

## Results

### Behavioral data

A repeated measures ANOVA for RTs including factors GROUP (PG vs. OG) * RISK (high-risk vs. low-risk) did not show any significant GROUP-related main effects, and GROUP * RISK interaction. Both groups showed significantly longer RTs in high-risk compared to low-risk task conditions (main effect of the factor RISK; 1999 ± 705 ms (mean ± SD) vs. 1578 ± 648 ms; F_[1,22]_ = 50.9; p < 0.001). Exclusion of the four problem gamblers (DSM IV score: 3 or 4) did not change the overall pattern of results (no significant interaction; main effect of the factor RISK; 1936 ± 630 ms (mean ± SD) vs. 1502 ± 602 ms; F_[1,22]_ = 50.2; p < 0.001). A repeated measures ANOVA for RTs including the factors group (PG vs. OG) * high-risk decision (high-risk hit vs. high risk stand) revealed no significant effects (also no significant effects in the analysis without the four problem gamblers). In the low-risk task condition only six (three PG and three OG) out of 24 participants showed stand trials, and therefore the respective RTs were not further analyzed. Furthermore, PG and OG did not differ in the number of bust trials (in case participants draw another card and get more than 21 points). The same analysis without problem gamblers revealed also no differences between groups. A repeated measures ANOVA for decision behavior including the factors group (PG vs. OG) * decision behavior (percent high-risk hit vs. percent low-risk hit) revealed a main effect of decision behavior. Both groups showed significantly lower percentage of high-risk compared to low-risk hit trials (F_[1,22]_ = 57.2, 58.33 ± 24.69% (mean ± SD) vs. 97.08 ± 6.27%; p < 0.001). Exclusion of the four problem gamblers also showed a main effect of the factor decision behavior; 59.9 ± 22.86% (mean ± SD) vs. 96.9 ± 6.7%; F_[1,22]_ = 55.8; p < 0.001).

### ERP data

**Figure 2:**
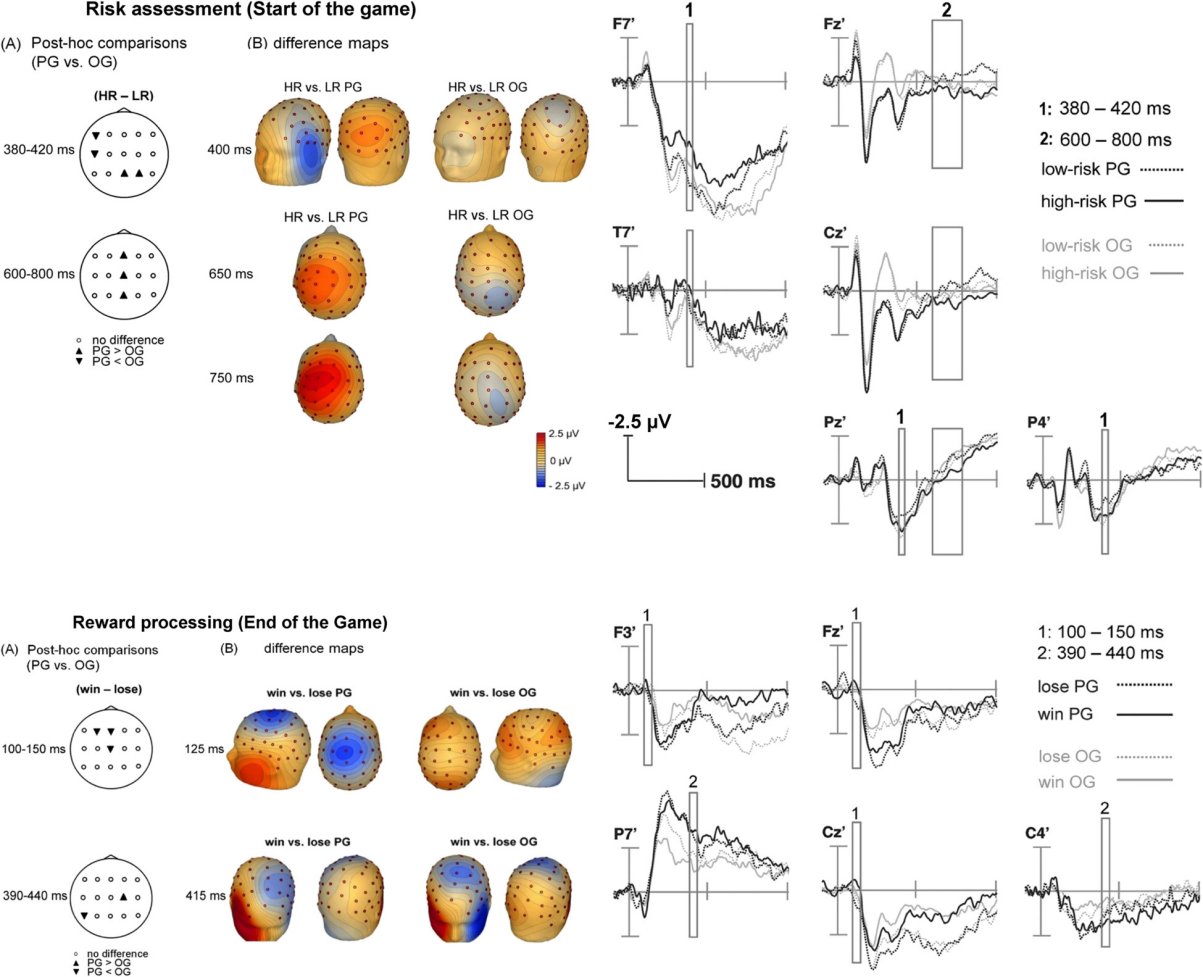
**ERP data. (A)** Significant differences of mean amplitudes in microvolt (μV): Post-hoc comparisons (high-risk (HR) vs. low-risk (LR); above, and win vs. lose; below) between groups at 15 electrode positions. Symbols in the underline represent the direction of significant differences (independent-sample-t-tests, p < .05). **(B)** Spherical spline maps (EEG-voltage, 0.25 μV/step) displaying difference maps per group for selected time points both during risk assessment and reward processing. ERPs are displayed to the right.

For risk assessment a four-way interaction (AP * LAT * RISK * GROUP) did not reveal any significant effect in any time window of interest. For the 380-420 ms time window, a three-way interaction (AP * RISK * GROUP), (F_[1.7,38.5]_ = 3.83, p = 0.041, Greenhouse-Geisser(GG)-adjusted; eta-squared(η^2^) = 0.12) was statistically significant, whereas the LAT * RISK * GROUP interaction showed a trend to significance (F_[2.5,56.0]_ = 3.05, p = 0.052, GG-adjusted; η^2^ = 0.12). Post-hoc tests indicated significantly lower mean amplitude values (difference: high-risk vs. low-risk) in PG compared to OG at F7 (t_[1,22]_ = 3.33, p = 0.003) and T7 (t_[1,22]_ = 2.73, p = 0.012) electrode locations, and significantly higher mean amplitude values (difference: high-risk vs. low-risk) in PG compared to OG at Pz (t_[1,22]_ = 2.38, p = 0.026) and P4 (t_[1,22]_ = 3.69, p = 0.001) (see Figure 2, upper part). For the late time window (600-800 ms) there was a significant LAT * RISK * GROUP interaction, (F_[2.5,54.8]_ = 3.89, p = 0.025, GG-adjusted; η^2^ = 0.14). Post-hoc tests indicated significantly higher mean amplitude values (difference: high-risk vs. low-risk) in PG compared to OG at midline electrodes Fz (t_[1,22]_ = 2.25, p = 0.035), Cz (t_[1,22]_ = 2.26, p = 0.034), and Pz (t_[1,22]_ = 3.33, p = 0.038; see Figure 2, upper part).

For reward processing a four-way interaction (AP * LAT * REWARD * GROUP) did not reach significance in any time window of interest. For the 100-150 ms time window there was a statistical trend for a three-way interaction (LAT * REWARD * GROUP; F_[2.4,51.9]_ = 2.69, p = 0.078, GG-adjusted; η^2^ = 0.10). Post-hoc tests indicated significantly lower mean amplitude values (difference: win vs. lose) in PG compared to OG at electrode positions F3 (t_[1,22]_ = 2.27, p = 0.033), Fz (t_[1,22]_ = 2.91, p = 0.008), and Cz (t_[1,22]_ = 2.27, p = 0.034; see Figure 2, lower part). For the 390-440 ms time window a three-way interaction (AP * REWARD * GROUP; F_[1.4,31.6]_ = 2.99, p = 0.085, GG-adjusted; η^2^ = 0.11) revealed a statistical trend. Applied post-hoc tests indicated significantly lower mean amplitude values (difference: win vs. lose) in PG compared to OG at electrode P7 (t_[1,22]_ = 2.35, p = 0.028), and higher amplitude values (difference: win vs. lose) in PG compared to OG at C4 electrode (t_[1,22]_ = 2.18, p = 0.041).

### fMRI constrained source analysis

**Figure 3:**
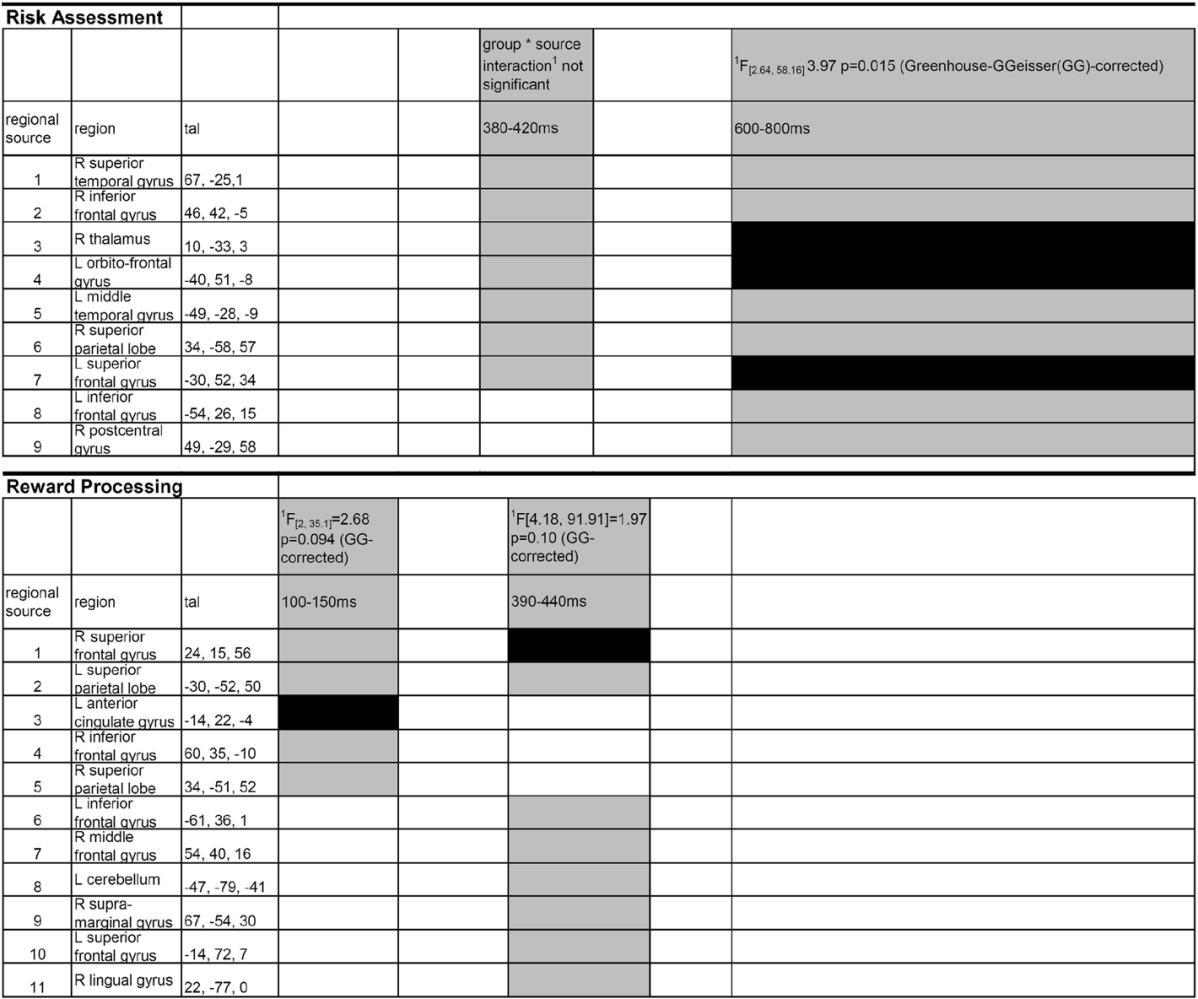
**Results of the fMRI-constrained source analysis.** Post-hoc t-tests, indicating significant differences between OG and PG for root mean square values of regional source moment activities revealed from fMRIconstrained source analyses applied on differences waves between high-risk and lowrisk, and win and lose trials discrete time windows related to ERP-effects (Figure 2). Sources which are included in the analysis according to the state of model fit are grey-shaded. Black boxes show trend to significant (regional source 3 at 100-150 ms during reward processing; p = 0.067) and significant (all other black boxes; p < 0.05) post-hoc t-tests.

For risk assessment discrete ANOVAs over relevant time windows according to ERP analysis demonstrated only significant effect of the group * RS interaction during the 600-800 ms time-window (see Figure 3 upper part). Post-hoc tests showed that PG demonstrated larger source moments than OG in the right thalamus, left orbitofrontal gyrus and left superior frontal gyrus.

For the phase of reward processing discrete ANOVAs over relevant time windows according to ERP analysis demonstrated significant or trend to significant effects of the GROUP * RS interaction (see Figure 3 lower part). Post-hoc tests revealed that PG compared to OG showed a statistical trend to enhanced source moments between 100 and 150 ms in left anterior cingulate gyrus followed by higher source activity in PG in the right superior frontal gyrus between 390-440 ms.

## Discussion

The present study aimed at investigating risk assessment and reward processing in PG with a combined EEG and fMRI approach to identify successively active brain regions in PG and OG during two phases of a blackjack game.

Although both groups showed the same pattern of behavior, ERP signals in PG and OG significantly differed in ERPs around 400 ms and in late time window on high-risk vs. low-risk decisions. The fMRI constrained regional source model during risk assessment demonstrated larger source moments in PG in the high-risk vs. low-risk comparison in thalamic, superior frontal and orbitofrontal activations during 600-800 ms. During reward processing as derived from contrasting winning vs. losing events, PG showed a trend to enhanced early fronto-central ERP amplitude and centro-parietal differences in late time window. There was a trend to higher source activity in an early time window in the ACC, followed by enhanced superior frontal (i.e., pre-motor-related) source activity in PG.

### Neural processing dynamics of risk assessment in OG and PG

PG compared to OG did not run a higher risk during the present blackjack game. However, slower RTs in high-risk compared to low-risk situations in both groups might be associated with heightened response conflict [44]. Moreover, both groups might have related their decisions to the same extent to the blackjack basis strategy [38].

PG compared to OG presented larger ERP mean amplitude differences between high-risk and low-risk task conditions at parietal electrode positions (Pz, P4) around 400 ms. Whereas PG showed higher amplitude in high-risk compared to low-risk trials, OG demonstrated the opposite pattern. A putative focus of OG on potentially safe low-risk situations accompanied by heightened amplitude in this time-window might reflect an elevated task relevance effect, and therefore more intense stimulus processing [45]. On the other hand, the enhanced amplitude in PG in high-risk relative to low-risk decisions might be related to enhanced intensity processing [45] as high-risk decisions seem to be more attractive than low-risk decisions in PG. Hence, high-risk situations related to physiological arousal and euphoria [46] might track enhanced attention in PG similar to observed effects in smokers during smoking cue processing [47]. Left frontal and left temporal electrode positions (F7 and T7) showed lower mean amplitude in PG compared to OG in the high-risk vs. low-risk difference wave around 400 ms. These effects were mainly caused by amplitude differences between high- and low-risk decisions exclusively in PG. The reduced left frontal positivity in PG high-risk compared to low-risk decision might reflect an indirect approach behavior [48] in appetitive high-risk situations in PG due to reduced inhibition.

Higher mean amplitude values in PG compared to OG in the high-risk vs. low-risk difference wave at central electrode positions (Fz, Cz, Pz,) during the slow wave time window was caused by higher late slow wave amplitude in high-risk situations than low-risk situations only in PG. Dolcos and Cabeza [49] reported increased positivity at fronto-central sites during recall of emotionally pleasant compared to unpleasant stimuli. Accordingly, PG might perceive high-risk situations as more pleasant since engagement in thrill seeking or impulsive behavior [50,51] offers PG the opportunity to compensate generally blunted reward processing [52] resulting from dopaminergic deregulation in mesolimbic brain structures [53]. On the other hand, PG’s intrinsic motivation during low-risk situations might have been weaker than in high-risk situations, which possibly led to a down-regulation of late slow wave amplitude [26] during motivationally irrelevant low-risk situations [54] probably driven by higher orbitofrontal and thalamic activity in PG as these regions have been reported to play an important role in cue-induced craving [55,56] and cue-induced urge for gaming [57]. Consequently, risk assessment in PG might be characterized by triggering of gambling concepts in frontal brain regions in combination with subcortical-driven affective craving processing.

### Neural processing dynamics of reward processing in OG and PG

A trend to increased negativity between 100 and 150 ms in PG compared to OG in the win vs. lose difference wave at fronto-central electrode positions (F3, Fz, Cz) resulted from more negative amplitudes in the win relative to the lose condition exclusively in PG, which was potentially driven by prior enhanced rostral cingulate activations in PG. These differences might be related to heightened attentional processing [31], addiction memory involvement, and heightened sensitivity for drug-cues [19]. As winning money is a relevant requirement for PG, enabling them to place the next bet to continue with gambling, and strengthening the desire to persist in gambling [58], our results are also in agreement with early fronto-central ERP modulation during biologically-relevant stimuli processing [59]. Source analysis data of the present study revealed a trend to higher rostral ACC activity in PG, which might point to enhanced affective processing of conditioned cues in PG, as McClernon [60] reported a positive correlation between pre-scan craving and smoking cue activation in rostral ACC, dorso-medial prefrontal cortex, SFG, and supplementary motor area in smokers.

A trend to higher mean amplitude values in PG compared to OG in the win vs. lose difference wave at right central electrode position (C4; between 390 and 440 ms) resulted from higher amplitude in win situations than lose situations in PG, whereas OG showed the opposite pattern. High arousal levels in PG might be coupled to win situations, whereas OG might have been aroused during losing money, both reflected by augmented amplitudes [32]. In addition, increased negativity in PG compared to OG in the win vs. lose difference wave at left parietal electrode position (P7) resulted from more negative amplitude in win situations than lose situations in PG, whereas OG showed the opposite pattern. Therefore, enhanced negativity of left parietal P7 electrode amplitude in PG might be related to strengthened attention or context updating in working memory [61] or long-term perceptual expertise [62] during win situations in PG, whereas OG might spend higher attention to monetary losses. Interestingly, stronger superior-frontal/pre-motor source activity in PG might indicate enhanced cue-based action selection [63] while winning real money preceded by early affective processing in rostral ACC.

### Limitations

The OG group was equally familiar with the experimental blackjack condition as gamblers without showing pathological gambling behavior, therefore they did not represent normal control participants as described in other studies. As PG were slot machine gamblers, the results of the present study cannot be generalized to pathological casino gamblers, which demonstrated less decision making deficits [64]. One reason why we did not find feedback-related negativity [11] might be derived from the fact that it was difficult to lock ERPs to the exact time-point when participants identified a win or a lose situation due to variable timespans needed for counting the points. In addition, the present study might be under-powered due to small sample size. Incorporating marginal significant effects might outweigh the gain of important information about underlying neuronal mechanisms of problem gambling obtained by the explorative and hypothesis generating character of our study.

## Conclusion

Taken together, source analyses impressively demonstrated the spatio-temporal dynamics of the differences between groups related to the underlying neural generators. On the one hand, risk assessment dominantly produced neocortical fronto-thalamic source activations in PG as compared to OG, suggesting top-down processing in risky situations in PG. On the other hand, during reward processing early rostral-cingulate and later neocortical frontal source activity suggest pronounced bottom-up processing in PG as compared to OG. Summarizing, risk assessment seems to be a rather cognitive process in PG, and reward processing might rather be processed emotionally in PG as compared to OG. Treatments of addictive gambling might address the adequate cognitive appraisal of risk and the appropriate emotional evaluation of context-related reward.

## Additional file

Additional file 1: Figure S1. Source model for risk assessment: (A) Electrode overlay plot of the high-risk vs. low-risk difference wave for PG minus OG (-200-1000 ms); (B) Global field power (GFP; blue curve) and residual variance [(RV) and best fit; red curve]; (C) Regional sources (RS) with Talairach coordinates and scalp location (L = left; R = right). Regional sources 1-3 were seeded according corresponding fMRI peak activations – RS 4 to 9 were added by sequential fitting procedures. **Figure S2.** Source model for reward processing: (A) Electrode overlay plot of the win vs. lose difference wave for PG minus OG (-200-1000 ms); (B) Global field power (GFP; blue curve) and residual variance [(RV) and best fit; red curve)]; (C) Regional sources (RS) with Talairach coordinates and scalp location (L = left; R = right). Regional sources 1 and 2 were seeded according corresponding fMRI peak activations – RS 3 to 11 were added by sequential fitting procedures. **Figure S3.** 15 approximately equidistant distributed electrode positions where topographical analyses of mean amplitude values were performed separately for each ERP time interval.
