# Supplementary material for: Risk assessment and reward processing in problem gambling investigated by event-related potentials and fMRI-constrained source analysis
Source: BMC Psychiatry. 2014 Aug 10;14:229. doi: 10.1186/s12888-014-0229-4 (PMC4149195; doi:10.1186/s12888-014-0229-4)
Supplement: Additional file 1: Figure S1. — Source model for risk assessment: (A) Electrode overlay plot of the high-risk vs. low-risk difference wave for PG minus OG (-200-1000 ms); (B) Global field power (GFP; blue curve) and residual variance [(RV) and best fit; red curve]; (C) Regional sources (RS) with Talairach coordinates and scalp location (L = left; R = right). Regional sources 1-3 were seeded according corresponding fMRI peak activations – RS 4 to 9 were added by sequential fitting procedures. Figure S2. Source model for reward processing: (A) Electrode overlay plot of the win vs. lose difference wave for PG minus OG (-200-1000 ms); (B) Global field power (GFP; blue curve) and residual variance [(RV) and best fit; red curve)]; (C) Regional sources (RS) with Talairach coordinates and scalp location (L = left; R = right). Regional sources 1 and 2 were seeded according corresponding fMRI peak activations – RS 3 to 11 were added by sequential fitting procedures. Figure S3. 15 approximately equidistant distributed electrode positions where topographical analyses of mean amplitude values were performed separately for each ERP time interval. [file 12888_2014_229_MOESM1_ESM.doc]

**Additional file 1:**


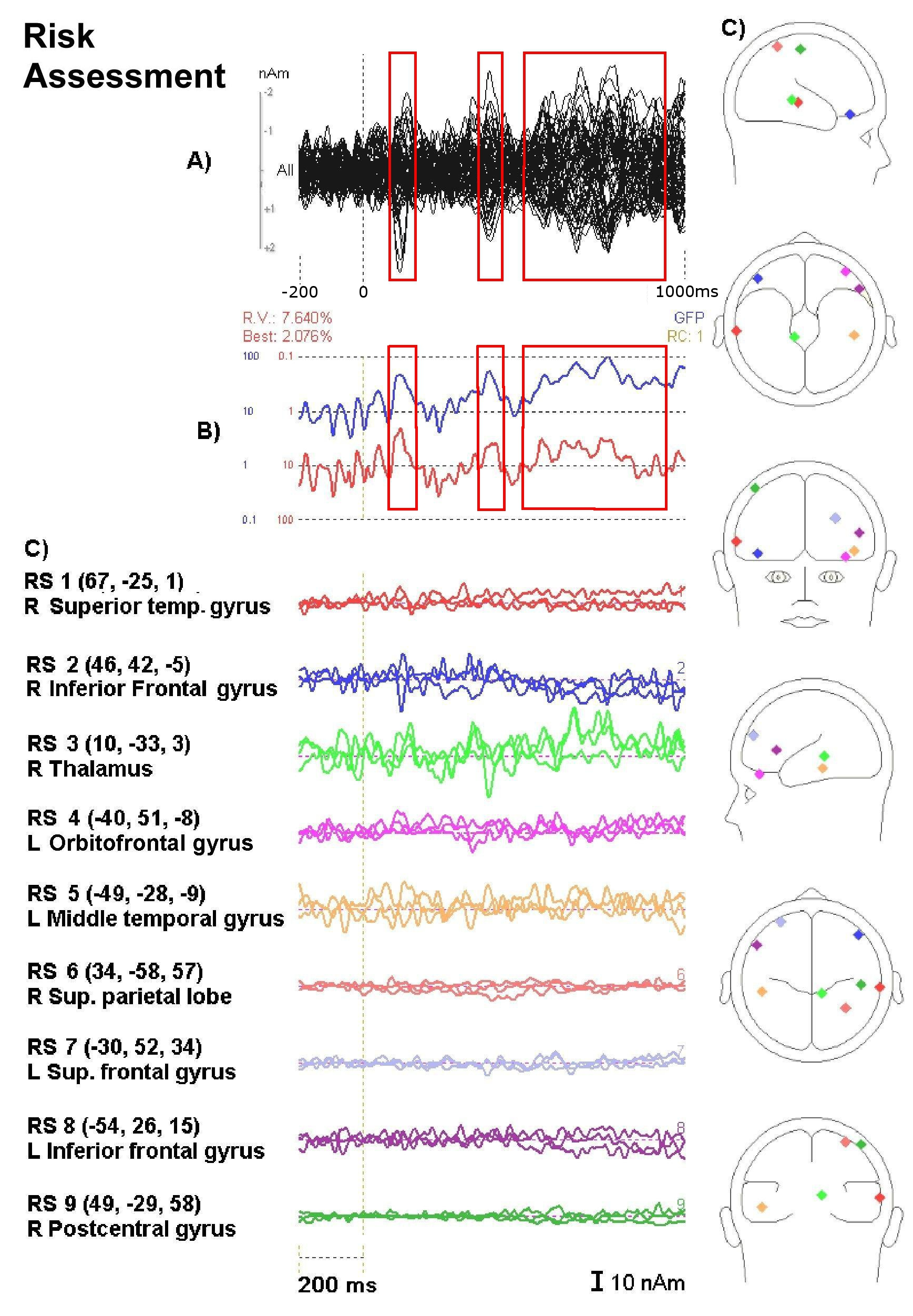


Figure S1. Source model for risk assessment: (A) Electrode overlay plot of the high-risk vs. low-risk difference wave for PG minus OG (-200-1000 ms); (B) Global field power (GFP; blue curve) and residual variance [(RV) and best fit; red curve]; (C) Regional sources (RS) with Talairach coordinates and scalp location (L=left; R=right). Regional sources 1-3 were seeded according corresponding fMRI peak activations – RS 4 to 9 were added by sequential fitting procedures.


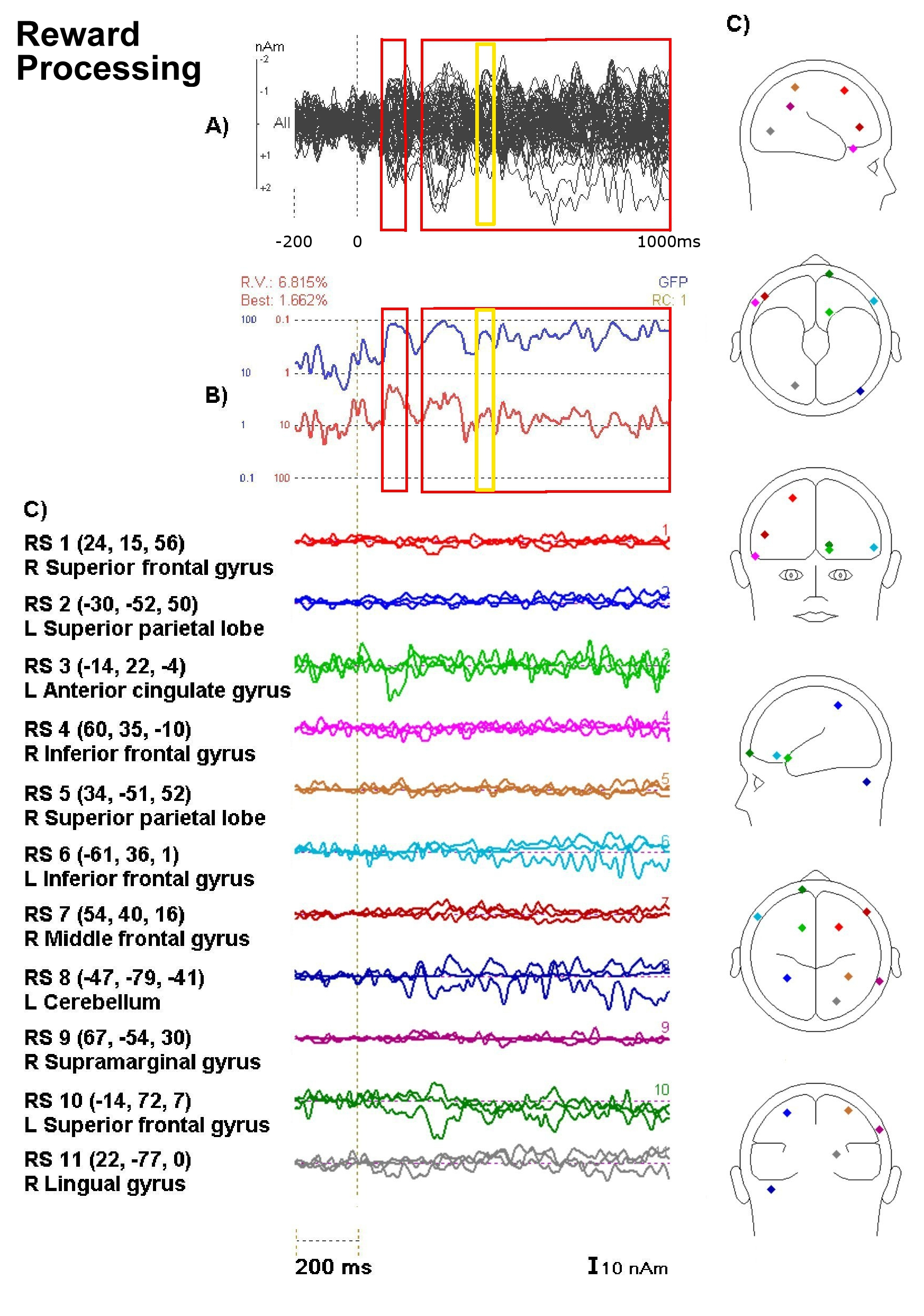


Figure S2. Source model for reward processing: (A) Electrode overlay plot of the win vs. lose difference wave for PG minus OG (-200-1000 ms); (B) Global field power (GFP; blue curve) and residual variance [(RV) and best fit; red curve)]; (C) Regional sources (RS) with Talairach coordinates and scalp location (L=left; R=right). Regional sources 1 and 2 were seeded according corresponding fMRI peak activations – RS 3 to 11 were added by sequential fitting procedures.


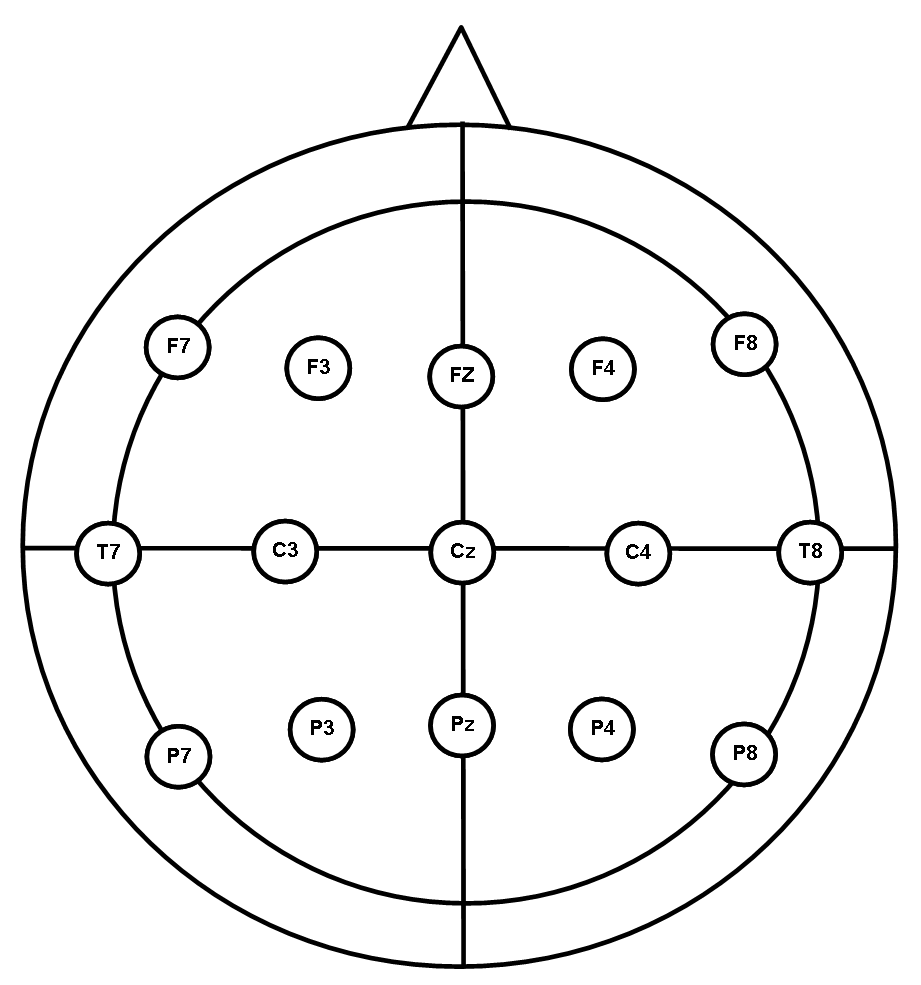


Figure S3. 15 approximately equidistant distributed electrode positions where topographical analyses of mean amplitude values were performed separately for each ERP time interval.
